# Acceptance of COVID-19 vaccine and associated factors among health care workers at public hospitals in Eastern Ethiopia using the health belief model

**DOI:** 10.3389/fpubh.2022.957721

**Published:** 2022-11-04

**Authors:** Tamirat Getachew, Magarsa Lami, Addis Eyeberu, Bikila Balis, Adera Debella, Bajrond Eshetu, Meron Degefa, Sinetibeb Mesfin, Abraham Negash, Habtamu Bekele, Getahun Turiye, Dawit Tamiru, Kabtamu Nigussie, Henock Asfaw, Yadeta Dessie, Addisu Alemu, Addisu Sertsu

**Affiliations:** ^1^School of Nursing and Midwifery, College of Health and Medical Sciences, Haramaya University, Harar, Ethiopia; ^2^School of Public Health, College of Health and Medical Sciences, Haramaya University, Harar, Ethiopia

**Keywords:** COVID-19, vaccine acceptance, vaccine hesitancy, health care workers, Ethiopia

## Abstract

**Introduction:**

Acceptance of COVID-19 vaccination among Health Care Workers is mandatory to lessen and curve the spread of transmission of COVID-19. Even though the Health Belief Model is one of the most widely used models for understanding vaccination behavior against COVID-19 disease, COVID-19 vaccine acceptance among Health Care Workers in Ethiopia was not adequately explored by using the Health Belief Model domains.

**Purpose:**

This study aimed to assess COVID-19 vaccine acceptance and associated factors among Health care workers in eastern, Ethiopia.

**Methods:**

Institutional-based cross-sectional study design was used among 417 health care workers selected by a systematic random sampling method from June 1- 30/2021. The data were collected by face-to-face interviews using semi-structured questionnaires and analyzed using STATA version 14 statistical software. Multivariable binary logistic regression analysis with a 95% confidence interval was carried out to identify factors associated with willingness to COVID-19 vaccine acceptance and a statistical significance was declared at a *P*-value < 0.05.

**Results:**

The willingness of health care workers to accept the COVID-19 vaccine was 35.6%. Age 30-39 (AOR = 4.16;95% CI: 2.51, 6.88), age ≥ 40 years (AOR = 3.29;95% CI: 1.47, 7.39), good attitude (AOR = 1.97; 95% CI: 1.00, 3.55), perceived susceptibility (AOR = 1.93; 95% CI: 1.12, 3.32), and perceived severity (AOR = 1.78; 95% CI: 1.03, 3.10) were factors significantly associated with Health Care Workers acceptance of COVID-19 vaccine.

**Conclusion:**

The willingness to accept the COVID-19 vaccine among HCWs was low. Factors significantly associated with the willingness to accept the COVID-19 vaccine were age, good attitude, perceived susceptibility, and perceived severity of the disease. The low willingness of Health Care Workers to accept the COVID-19 vaccine was alarming and it needs more emphasis from the government in collaboration with other stakeholders to provide reliable information to avert misconceptions and rumors about the vaccine to improve the vaccine status of Health Care Workers to protect the communities.

## Introduction

The Coronavirus (COVID-19) pandemic is a public health concern, and there are no particular antiviral medicines available for COVID-19 at this time (1–3). The COVID-19 pandemic is projected to continue to wreak havoc on society and economies around the world, causing massive morbidity and mortality (4). Health Care workers (HCWs) are the primary responsible person for controlling COVID-19 and are at higher risk of contracting the virus (5). Health Care Workers (HCWs) Susceptibility to diseases like COVID-19 has several consequences particularly in low-income nations by limiting the number of HCWs which may result in crises in healthcare systems. Moreover, health professionals are always frontline with the case and frequently contact clients, they have the potential to infect others (6).

To achieve optimal vaccine coverage and avert ongoing public spread, COVID-19 control will most likely rely on successful vaccine development and distribution to a large segment of the population. Unprecedented efforts have been made to develop COVID-19 vaccinations to combat the pandemic (7). Several vaccines have been approved for use as early as the end of 2020 in Canada and the European Union since December 2020 (8, 9). All countries are battling the spread of COVID-19 with quarantine and lockdowns, social distancing measures, public usage of facemasks, and travel restrictions until vaccinations or effective treatments become available (10, 11). An effective vaccination, in combination with protective measures, will be the most effective strategy for mitigating the spread of COVD-19 and promoting positive clinical and socioeconomic consequences (12).

COVID- 19 vaccinations are now accessible, and many countries, including Ethiopia, have already reserved supplies of the long-awaited vaccines. Any vaccination program's success, however, is contingent on high vaccine acceptability and uptake, and the fundamental problem presently facing the public is instilling public faith in an emergency-released vaccine. Vaccine acceptance is on the verge of becoming a reality without such assurance (13, 14).

Despite the enormous efforts made to develop viable COVID-19 vaccines, vaccine acceptability toward the approved and projected COVID-19 immunization remains a serious roadblock (14). COVID-19 vaccine reluctance among HCWs may be comparable to rates in the general population, according to evidence (15); a meta-analysis study revealed that only 51% of HCWs were willing to get the vaccine (16). The complacency of not getting infected, lack confidence in the vaccine and vaccination service system's safety and effectiveness, the ease of seeking service, and higher-than-expected costs may all contribute to a reduction in the likelihood of receiving the vaccination (17, 18).

In nations like Ethiopia, where the healthcare system is characterized by limited surveillance and laboratory capability, a paucity of healthcare human resources, and insufficient financial capacity, an outbreak of a cureless viral infection with no vaccination would be disastrous (18). Despite the Ethiopian government's significant initiatives and recognition of COVID-19's public health value (screening, quarantine, and treatment centers), there is a pressing need to increase HCWs' willingness to adopt the COVID-19 vaccine (19, 20). Numerous research demonstrated the value of interventions focusing on health belief model (HBM) constructs for boosting vaccination uptake (21, 22) and it's one of the most often employed models used for understanding vaccination behavior against COVID-19 (22, 23). According to this theory, many variables like perceived susceptibility, perceived severity, perceived benefits, perceived barriers, and cues to action influence the health-related behavior of individuals (24). Perceived susceptibility refers to perceptions of vulnerability to infection while Perceived severity refers to perceptions of the consequences of catching the infection. Perceived benefits and perceived barriers are terms used regarding vaccination; the former refers to a person's beliefs about getting immunized, while the latter refers to the notion that getting immunized is constrained by psychosocial, physical, or financial factors. Information, people, and events that direct or guide an individual to be vaccinated are examples of cues to action (25, 26).

The finding from previous and recent studies are showing that COVID-19 vaccination has substantially altered the course of the pandemic, saving tens of millions of lives globally (27). The global morbidity and death caused by COVID-19 are becoming reduced due to the wide distribution of COVID-19 immunization (28). However, unwillingness toward the vaccine is becoming a challenge and barrier to covering a large proportion of the vulnerable population, estimates of vaccine acceptance among HCWs were scarce and not addressed using the health belief model yet in Ethiopia. To the best of our knowledge, this is the first study that uses HBM components to assess the acceptance of the COVID-19 vaccine among HCWs. To do so, it's crucial to assess the HCWs level of vaccination acceptability of COVID-19 to combat the virus pandemic effects. Therefore, this study aimed to assess COVID-19 vaccination acceptance and associated factors among HCWs working in public hospitals in Eastern Ethiopia using the health belief model so that public health experts and the government could target the most vulnerable communities.

## Materials and methods

### Study design, setting, and period

An institutional-based cross-sectional study design was conducted among seven randomly selected public hospitals in eastern Ethiopia (Dilchora, Bisidimo, Haramaya, Gara Muleta, Deder, Chiro, and Gelemso hospitals) from June 1- 30/2021. Dilchora hospital is a referral hospital found in Dire Dawa city administration which gives comprehensive health services for both urban and rural populations surrounding the city. East Hararghe has a total population of 3,587,042. West Harerghe zone has a total population of 2,467,364.

### Study population

The source populations were all HCWs who were working in public hospitals in Eastern Ethiopia. The study populations were all HCWs who were working in selected public hospitals in Eastern Ethiopia during the study period.

### Eligibility criteria

All HCWs who were on duty during data collection, and have willing to participate in the study were included in the study. HCWs who were on annual leave, maternal leave, and sick leave during the study period were excluded.

### Sample size determination and sampling procedure

The required sample size was determined by using the single population proportion formula (n = (Z_α/2_)^2^*p* (1-*p*)/ d2) with the following assumptions: the prevalence of COVID 19 vaccine acceptance among healthcare workers (*p* = 56%), from a study conducted in the Democratic Republic of Congo (29); Confidence level at 95% (Z_α/2_) = 1.96, a margin of error (d) = 0.05 and non-response rate = 10%. So, the final sample size was 417. Seven public hospitals (Dilchora, Bisidimo, Haramaya, Gara Muleta, Deder, Chiro, and Gelemso hospital) found in the study area were randomly selected and included in the study. About 320, 182, 164, 142, 127, 108, and 102 HCWs were found in the hospitals listed above, respectively. The required study samples from each public hospital were allocated proportionally to the size of HCWs of each Hospital. The study subjects were selected using a systematic random sampling technique with (K = N/n, = 1145/416 = 2.75 = 3) based on staff registration for HCWs until the predetermined sample size was obtained. The first eligible study participant was selected randomly.

### Measurement of variables

The data were collected by face-to-face interviews using a self-administered semi-structured questionnaire which was adapted from previous literature and some modification was made to suit the local context. The questionnaire contains four parts; which was designed to collect information on socio-demographic characteristics, COVID-19 Vaccine acceptance and health-related status of study participants, HCWs attitude toward the COVID-19 vaccine, and health belief measures using the Health Belief Model domain (30–32) and health belief measures using Health Belief Model (33).

In the sociodemographic characteristics section, personal details, including age, sex marital status, educational level, type of profession, number of family members, and monthly income, were queried. The HCWs' acceptance of the COVID-19 vaccin**e** was measured by asking a single item “Will you take the COVID-19 vaccine when it becomes available?” with ‘Yes’, and ‘No’ response options. If the respondents' answered ‘’yes”, he/she is considered as having the willingness to accept the COVID-19 vaccine and otherwise no (16). Again, HCWs were also asked if they were frontline workers, had an existing chronic disease, had ever been diagnosed with a chronic disease, anybody aged > 64 years old in their family, and anybody diagnosed with chronic disease in their family.

HCWs attitude toward the COVID-19 vaccine was determined based on 10 attitude assessment questions. Each question score was based on a five-point Likert scale, in which a score of 1 to 5 was given from strongly disagree to strongly agree. Then, the score was computed with a total minimum score of 10 and a maximum score of 50. A mean score was calculated for the computed value and a score below the mean was considered as having a poor attitude and a score above the mean value was described as having a good attitude toward the COVID-19 vaccine (32).

The Health Belief Model (HBM) was composed of five dimensions, including perceived susceptibility, perceived severity, perceived benefits, perceived barriers, and cues to action. The perceived susceptibility domain consisted of five items addressing HCW's sights about their possible risk of getting infected by COVID-19; I am susceptible to being infected due to my occupational exposure, COVID-19 infection is a very real possibility, People who are in good health can get COVID-19, COVID-19 is more likely to infect me because of my health, and I don't think I'll be able to protect myself from COVID-19 any better than other individuals. The perceived severity domain also consisted of five items to address HCWs' concerns about the seriousness of COVID-19.; COVID-19 has the potential to make some people severely sick and fatal, COVID-19 is more dangerous than the seasonal flu, I'll be unwell if I acquire COVID-19, If I contract COVID-19, I may need to be admitted to the hospital, I might die if I acquire COVID-19.

The perceived benefits domain consisted of six items to address perceived positive outcomes of getting vaccinated against COVID-19 in terms of reducing their susceptibility to contracting the illness or the severity of symptoms if being infected by COVID-19; Vaccination is a fantastic idea because it reduces my fear of contracting COVID-19, COVID-19 and its consequences are less likely to affect me if I am vaccinated, I safeguard my patients, family, and acquaintances from infection by being vaccinated, When I am vaccinated, the entire community benefits because COVID-19 is prevented from spreading, COVID-19 vaccine is a powerful tool for preventing and controlling the COVID-19 virus, and to stop the COVID-19 pandemic, high vaccine coverage is essential all across the world.

Perceived barriers domain consisted of thirteen items to address the HCWs concerns or negative beliefs toward COVID-19 vaccines; I am concerned about the COVID-19 vaccine's side effects, concerned about the COVID-19 vaccine's efficacy, concerned about the COVID-19 vaccine's safety, Concerned about the COVID-19 vaccine's price, Concerned about the vaccine's novelty, Concerned about the COVID-19 vaccine's availability, Concerned about the limited availability of the COVID-19 vaccination, Concerned about the halal status of the vaccines offered, Concerned about the manufacturer's and supply source's reliability, Concerned about the Ethiopian health system and vaccination distribution strategy, Concerned about the vaccine's administration mode (needles use), Concerned about the frequency of vaccines (number of doses required), and Concerned about the longevity of immunity (how much time I will be protected).

The cues to action domain included six items to address different clues or recommendations that promote the willingness of HCWs to get vaccinated against COVID-19.; Once credible information is provided, COVID-19 vaccination uptake, if health facilities recommend it, the COVID-19 vaccine up-taken, If the COVID-19 vaccination is recommended by the health authorities, it will up-taken, If the media recommends the COVID-19 vaccination, it will be accepted, if my work recommends it, I will get the COVID-19 vaccine, and if a large number of people get the COVID-19 vaccination, it will be accepted.

Respondents were asked to rate all HBM items on a five-point scale ranging from 1 (strongly disagree) to 5 (strongly agree). A total score for each dimension was computed and the mean score for each domain was calculated. Higher scores (above the mean) indicate greater levels of a specific domain of dimension except for the perceived barrier domain which was reversely coded (higher perceived barrier scores indicated lower levels of perceived barriers).

### Data collection procedures

After full informed consent was obtained from each study participant, the data were collected by 2 diploma nurses and 2 midwives who are not working in the study area and supervised by four BSc holder nurses. A brief introductory orientation was given to the study participants by data collectors about the purposes of the study and the importance of their involvement. Then, HCWs who were volunteers were interviewed using semi-structured and pre-tested questionnaires. The data was collected for the duration of 1 month from June 1-30/021.

### Data quality control

Before beginning actual data collection, a pretest was done at the local public hospital (Jegula hospital) on 5% of the sample size (21 HCWs). The pre-test findings and experiences were used to improve and reshape the data collection tools. Before data collection, data collectors and supervisors received training on the study's goal, the confidentiality of information, and how to respect respondent rights and privacy. The investigators evaluated the completed questionnaires for completeness, accuracy, and clarity of data, and any necessary modifications were made immediately by the principal investigator and supervisors daily.

### Data management and analysis

The data was collected with the Kobo Collect software version 2021.3.4 and then exported to STATA version 14 for analysis. Participants' socio-demographic characteristics, awareness of COVID-19, attitude toward the COVID-19 vaccine, and health belief measures utilizing HBM were described using descriptive statistical analyses such as simple frequency, mean, and standard deviation. Categorical variables were summarized using frequency and proportions, whereas continuous variables were summarized using mean and standard deviation. The data was then displayed using tables and frequencies. Collinearity was determined using VIF and tolerance, and the goodness of fit was determined using the Hosmer-Lemeshow statistic and the Omnibus test. The model was considered fit since it is found to be insignificant at p < 0.05. The chi-square test was used in the bivariate section while binary logistic regression was used to determine the factors that predict vaccination. Binary logistic regression was used because the dependent variable is dichotomous (HCWs vaccine acceptance classified as yes or no). In the multivariate analysis, the strength of statistical association for COVID-19 vaccine acceptance was assessed, along with the 95 percent confidence interval. Finally, a *p*-value of < 0.05 was declared statistically significant.

### Ethical consideration

Haramaya University's, College of Health and Medical Sciences, Institutional Health Research Ethics Review Committee (IHRERC) (reference number IHRERC/069/2021), provided ethical approval for this study. A letter of permission and support were provided to the selected seven public hospitals in which the study was carried out. Informed, voluntary, written, and signed consent was taken from the heads of each public hospital. Before the interview, each study participant gave their informed, voluntary, written, and signed consent, and they were offered the right to refuse or terminate the interview at any moment. Confidentiality of participants was maintained at all levels of the study throughout the data collection process. During data collection, the COVID-19 prevention protocol was strictly followed. There was no direct contact with patients and anonymity was maintained by using the identified number instead of the patient's name. Besides, the confidentiality of the data was kept and used for the study purpose only.

## Results

### Socio-demographic characteristics

Out of 417 study participants involved in this study, 416 HCWs were included in the final analysis making a response rate of 99.7%. The mean and standard deviation ages of study participants were 31 ± 17.24, respectively. The ratio of males to females was 0.97 to 1. About 288 (69.2%) HCWs have <5 years of experience and 233 (56.0%) have earned a salary of >6,000 Ethiopian birrs. Regarding the educational level, 281 (67.5%), and 35 (8.4%) HCWs were first-degree and third-degree (specialty) holders, respectively. Of the total HCWs participated in the study, 179 (43.0%) and 18 (4.3%) were Nurse and Anesthetist professionals, respectively (Table 1).

**Table 1:** Socio-demographic characteristics of HCWs working in public hospitals in Eastern Ethiopia, 2021 (*n* = 416).

**Variable**	**Category**	**Frequency**	**Percentage (%)**
Age	20–29	181	43.5
	30–39	197	47.4
	>40	38	9.1
Sex	Male	205	49.3
	Female	211	50.7
Marital status	Married	234	56.3
	Single	156	37.5
	Divorced	14	3.4
	Separated	10	2.4
	Widowed	2	0.5
Educational level	Diploma	53	12.7
	Degree	281	67.5
	Masters	47	11.3
	Third-degree (specialty)	35	8.4
Type of profession	Doctor	35	8.4
	Nurse	179	43.0
	Midwifery	96	23.1
	Pharmacist	36	8.7
	Laboratory	25	6.0
	Psychiatry nurse	27	6.5
	Anesthetist	18	4.3
Number of family members	1	24	5.8
	2	68	16.3
	>3	324	77.9

### COVID-19 vaccine acceptance and health-related status health care workers

Of the total study participants, 147(35.3%) HCWs have a willingness to accept the COVID-19 vaccine. Unreliability of the vaccine due to its development within a short period of time 122 (45.4%), fear of side effects 102 (37.9%), and doubts about the vaccine 88 (32.7%) were some of the reasons for the non- acceptance of the vaccine. Out of the total study participants, 71 (17.1%) of them were frontline workers and 48 (11.5%) had an existing chronic disease (Table2).

**Table 2:** COVID-19 vaccine acceptance and health-related status of HCWs working in public hospitals in Eastern Ethiopia, 2021 (*n* = 416).

**Variable**	**Category**	**Frequency**	**Percentage (%)**
Willingness to accept COVID-19 vaccine	**Yes**	**147**	**35.3**
	No	269	64.7
If no, the reason for not taking the vaccine (multiple answers) (*n* = 269)	Fear of side effects	102	37.9
	It's a biological weapon	22	8.2
	Doubts about vaccine	88	32.7
	Unreliable due to short time development	122	45.4
	Not enough information	22	8.2
	The vaccine itself causes COVID-19	11	4.1
	Ineffective	7	2.6
	No vaccine is needed (Covid-19 is overrated)	6	2.2
Have you ever been diagnosed with a chronic disease	Yes	50	12.0
	No	366	88.0
Anybody aged > 64 in above in your family	Yes	80	19.2
	No	336	80.8
Anybody diagnosed with chronic disease in your family	Yes	54	13.0
	No	362	87.0

### The attitude of HCWs toward the Covid-19 vaccine

The mean score of attitude-related questions was 36.2 with a standard deviation of 5 ± 34. Among the total study participants, 216 (51.9%) HCWs have a positive attitude toward the Covid-19 vaccine. Of the total study participants, 15 (3.6%), 49 (11.8%), 135 (32.5%), 159 (38.2%), and 58 (13.9%) of study participants strongly disagreed, disagree, neutral, agree and strongly agreed with the idea of COVID-19 can be avoided with vaccination. About 9 (2.2%), 28 (6.7%), 31 (7.5%), 169 (40.6%) and 179 (43.0%) study participants strongly disagree, disagree, neutral, agree and strongly agree with the idea of COVID-19 vaccination is necessary for health care workers (Table3).

**Table 3:** Attitude of HCWs toward COVID-19 vaccine among health care workers working in public hospitals in Eastern Ethiopia, 2021 (*n* = 416).

**Variable**	**Strongly disagree (%)**	**Disagree (%)**	**Neutral (%)**	**(Agree (%)**	**Strongly (agree (%)**
Do you believe COVID-19 can be avoided with vaccination?	15(3.6)	49(11.8)	135(32.5)	159(38.2)	58(13.9)
Do you think the currently available vaccine will be effective in preventing COVID-19 infection?	2(0.5)	74(17.8)	80(19.2)	169(40.6)	91(21.9)
Do you believe the COVID-19 vaccine, which has been granted a license, has been thoroughly tested in clinical trials?	15(3.6)	49(11.8)	134(32.2)	160(38.5)	58(13.9)
Do you believe that COVID-19 vaccination should be necessary for health care workers?	9(2.2)	28(6.7)	31(7.5)	169(40.6)	179(43.0)
Do you think the COVID-19 vaccine that is now available is effective?	14(3.4)	51(12.3)	138(33.2)	157(37.7)	56(13.5)
Are you confident in the safety of the present COVID-19 vaccine?	7(1.7)	52(12.5)	138(33.2)	164(39.4)	55(13.2)
Do you trust the advice of professionals?	15(3.6)	49(11.8)	135(32.5)	159(38.2)	58(13.9)
Do you trust the vaccine information disseminated by the official media?	66(15.9)	117(28.1)	127(30.5)	72(17.3)	34(8.2)
Do you believe the information supplied by the Ethiopian Public Health authority about COVID-19 vaccination is accurate?	24(5.8)	44(10.6)	82(19.7)	172(41.3)	94(22.6)
Do you think the COVID-19 vaccination will be affordable and accessible for all populations?	19(4.6)	120(28.8)	82(19.7)	125(30.0)	70(16.8)

### Health believes model measures

Two hundred sixty-nine (64.7%) and two hundred forty-one (57.9%) HCWs were scored above the calculated mean value on perceived susceptibility (α = 0.82) and severity domain (α = 0.71) of the HBM domain, respectively. Again, two hundred sixty (62.5%) and two hundred three (48.8%) HCWs scored above the mean value on perceived benefit (α = 0.89) and barrier (α = 0.76) of the HBM domain, respectively. Regarding the cues to action (α = 0.84) domain of HBM, 262 (63%) HCWs scored above the mean value (Table 4).

**Table 4:** HBM items: Perceived susceptibility, perceived severity, and seriousness, perceived benefits, perceived barriers, and cues of action status of HCWs working in public hospitals in Eastern Ethiopia, 2021 (*n* = 416).

**Variable**	**Strongly disagree (%)**	**Disagree (%)**	**Neutral (%)**	**Agree (%)**	**Strongly agree (%)**
Perceived susceptibility					
I am susceptible to being infected due to my occupational exposure	14(3.4)	69(16.6)	72(17.3)	170(40.9)	91(21.9)
COVID-19 infection is a very real possibility	20(4.8)	67(16.1)	62(14.9)	186(44.7)	81(19.5)
People who are in good health can get COVID-19	5(1.2)	14(3.4)	24(5.8)	227(54.6)	146(35.1)
COVID-19 is more likely to infect me because of my health	4(1.0)	6(1.4)	16(3.8)	214(51.4)	176(42.3)
I don't think I'll be able to protect myself from COVID-19 any better than other individuals	10(2.4)	11(2.6)	39(9.4)	225(54.1)	131(31.5)
Perceived severity and seriousness					
COVID-19 has the potential to make some people severely sick and fatal	5(1.2)	7(1.7)	10(2.4)	239(57.5)	155(37.3)
COVID-19 is more dangerous than the seasonal flu	34(8.2)	54(13.0)	36(8.7)	142(34.1)	150(36.1)
I'll be unwell if I acquire COVID-19.	91(21.9)	137(32.9)	37(8.9)	85(20.4)	66(15.9)
If I contract COVID-19, I may need to be admitted to the hospital	7(1.7)	46(11.1)	84(20.2)	181(43.5)	98(23.6)
I might die if I acquire COVID-19	8(1.9)	52(12.5)	136(32.7)	150(36.1)	70(16.8)
Perceived benefits					
Vaccination is a fantastic idea because it reduces my fear of contracting COVID-19.	12(2.9)	37(8.9)	74(17.8)	172(41.3)	121(29.1)
COVID-19 and its consequences are less likely to affect me if I am vaccinated.	12(2.9)	55(13.2)	78(18.6)	186(44.7)	85(20.4)
I safeguard my patients, family, and acquaintances from infection by being vaccinated.	13(3.1)	34(3.2)	27(6.5)	187(45.0)	155(30)
When I am vaccinated, the entire community benefits because COVID-19 is prevented from spreading.	5(1.2)	25(6.0)	39(9.4)	226(54.3)	121(29.1)
COVID-19 vaccine is a powerful tool for preventing and controlling the COVID-19 virus	7(1.7)	40(9.6)	71(17.1)	181(43.5)	117(28.1)
To stop the COVID-19 pandemic, high vaccine coverage is essential all across the world.	4(1.0)	26(6.3)	79(19.0)	205(49.3)	102(24.5)
Perceived barriers					
Concerned about the COVID-19 vaccine's side effects	78(18.8)	101(24.3)	68(16.3)	100(24.4)	69(16.6)
Concerned about the COVID-19 vaccine's efficacy	80(19.2)	116(27.9)	28(6.7)	99(23.8)	93(22.4)
Concerned about the COVID-19 vaccine's safety	60(14.4)	100(24.0)	72(17.3)	99(23.8)	85(20.4)
Concerned about the COVID-19 vaccine's price? (Willingness to pay)	62(14.9)	61(14.7)	22(5.3)	139(33.4)	132(31.7)
Concerned about the vaccine's novelty (not used before).	56(13.5)	67(16.1)	73(17.5)	137(32.9)	83.0(20.0)
Concerned about the COVID-19 vaccine's availability	72(17.3)	184(44.2)	50(12.0)	57(13.7)	53(12.7)
Concerned about the limited availability of the COVID-19 vaccination	65(15.6)	204(49.0)	57(13.7)	48(11.5)	42(10.1)
Concerned about the halal status of the vaccines offered	64(15.4)	54(13.0)	74(17.8)	136(32.7)	88(21.2)
Concerned about the manufacturer's and supply source's reliability	9(2.2)	64(15.4)	75(18.0)	178(42.8)	90(21.6)
Concerned about the Ethiopian health system and vaccination distribution strategy	55(13.2)	179(49)	53(12.7)	68(16.3)	61(14.7)
Concerned about the vaccine's administration mode (needles use).	7(1.7)	52(12.5)	138(33.2)	164(39.4)	55(13.2)
Concerned about the frequency of vaccines (number of doses required)	14(3.4)	174(41.8)	85(20.4)	94(22.6)	49(11.8)
Concerned about the longevity of immunity (how much time I will be protected)	30(7.2)	211(50.6)	59(14.1)	74(17.7)	43(10.3)
Cues to action					
Once credible information is provided, COVID-19 vaccination uptake	15(3.6)	50(12.0)	134(32.2)	159(38.2)	58(14.0)
If health facilities recommend it, the COVID-19 vaccine up-taken.	2(0.5)	74(17.8)	80(19.2)	169(40.6)	91(21.9)
If the COVID-19 vaccination is recommended by the health authorities, it will up-taken	7(1.7)	52(12.5)	138(33.2)	164(39.4)	55(13.2)
If the media recommends the COVID-19 vaccination, it will be accepted	15(3.6)	49(11.8)	135(32.5)	159(38.2)	58(13.9)
If my work recommends it, I will get the COVID-19 vaccine.	2(0.5)	74(17.8)	80(19.2)	169(40.6)	91(21.9)
If a large number of people get the COVID-19 vaccination, it will be accepted.	7(1.7)	52(12.5)	138(33.2)	164(39.4)	55(13.2)

### Factors associated with COVID-19 vaccine acceptance

In the bivariate analysis, factors like age, sex, diagnosis with chronic disease, experienced Covid-19 infection, frontline workers, attitude, perceived susceptibility, perceived severity, perceived benefits, and cues to action were associated with COVID-19 vaccine acceptance. But in the multi-variable logistic regression only age (30-39 and >40 years old), attitude, perceived susceptibility, and perceived severity were significantly associated with COVID-19 vaccine acceptance.

HCWs who were 30–39 years were more likely (AOR = 4.16; 95% CI:2.51, 6.88) to accept the COVID-19 vaccine compared with those aged 20–29 years. Again, HCWs who were >40 years were more likely (AOR = 3.29; 95% CI:1.47, 7.39) to accept the COVID-19 vaccine compared to those aged 20–29 years. HCWs who had a good attitude were (AOR = 1.97; 95% CI: 1.00, 3.55) more likely to accept the COVID-19 vaccine compared to those who had a poor attitude toward the COVID-19 vaccine. Perceived susceptibility predicted the willingness to accept the COVID-19 vaccine by 1.93 (AOR = 1.93, 95% CI: 1.12, 3.32) whereas perceived severity and seriousness of the disease predict the willingness to accept the COVID-19 by 1.79 (AOR = 1.79; 95% CI: 1.03, 3.10) (Table5).

**Table 5:** Factors associated with COVID-19 vaccine acceptance among HCWs Working in Public Hospitals in Eastern Ethiopia, 2021 (*N* = 416).

**Variable**	**Category**	**COVID-19 vaccine acceptance**		**COR (95% CI)**	**AOR (95% CI)**
		**Yes**	**No**		
Age	20–29	35	146	1	1
	30–39	95	102	3.89(2.45,6.17)	4.16(2.51,6.88)^**^
	>40	17	21	3.38(1.61,7.07)	3.29(1.47,7.39)^**^
Sex	Male	80	125	1.38(0.92,2.06)	1.48(0.93,2.35)
	Female	67	144	1	1
Diagnosed with chronic disease	Yes	18	32	1.03(0.56,1.91)	1.88(0.86,4.13)
	No	129	237	1	1
Experienced COVID-19 infection	Yes	23	25	1.81(0.99,3.32)	0.72(0.31,1.70)
	No	124	244	1	1
Frontline workers	Yes	31	40	1.53(0.91,2.57)	0.51(0.24,1.07)
	No	116	229	1	1
Attitude	Good	99	117	2.68(1.76,4.08)	1.97(1.00,3.55)^*^
	Poor	48	152	1	1
Perceived susceptibility	Yes	114	155	2.54(1.61,4.01)	1.93(1.12,3.32)^*^
	No	33	114	1	1
Perceived severity	Yes	107	134	2.60(1.75,4.16)	1.79(1.03,3.10)^*^
	No	40	135	1	1
Perceived benefits	Yes	106	154	1.93(1.25,2.98)	1.14(0.66,1.99)
	No	41	115	1	1
Cues to action	Yes	60	94	1.28(0.85,1.94)	0.57(0.33,0.98)
	No	87	175	1	1

* p < 0.05 and

** with p < 0.001; CI, Confidence Interval; COR, Crude Odd Ratio; AOR, Adjusted Odd Ratio.

## Discussion

The Willingness to accept the COVID-19 vaccine among HCWs was 35.6%. This study finding was in line with the study finding done in Ghana (34), and the United States (36%) (35), But the finding was higher than the study finding done in the general population of Ethiopia (31.4%) (36), and the democratic republic of Congo (27.7%) (37). The possible reason might be the time difference since the information about the COVID-19 vaccine was disseminated rapidly through various social media. Another reason might be there is a study population difference; the general population was the study population for a study done in Ethiopia while this study only done among healthcare workers.

However, the result of the study was significantly lower than the studies done in Ethiopia: in Southern Ethiopia (48.4%) (6), Gondar Zone Hospitals (45.3%) (30), and also studies conducted abroad in Beirut Lebanon (58%) (38), in China (39) in Pakistan (70.25%) (40), and in France (76.9%) (40). This difference might be due to study population differences, and differences in seriousness of the pandemic among different communities. Another reason might be there is varied information and doubts on social media about the vaccine. Furthermore, the low prevalence of willingness to accept COVID-19 acceptance might be explained by distribution of misinformation about the poor quality of the vaccine through mass Medias, and also there was rumors circulating in the healthcare providers about the vaccine side effects which made healthcare providers developed negative attitude, and affect their willingness to take COVID-19 vaccine.

Furthermore, the negative effects of social media and the propagation of misinformation regarding the vaccine's quality could explain the low acceptance (41, 42). The HCWs may have developed vaccine hesitation as a result of the widespread dissemination of disinformation and rumors about poor vaccine quality in the media, which may have influenced their decisions to accept vaccination and to promote the vaccine to their clients and the entire community.

Those HCWs whose age was found within the age category 30–39 and >40 years old were more likely to accept the COVID-19 vaccine compared to those found within the age category 20-29 respectively. This finding was in line with studies done in Hospitals of South Gondar Zone, Ethiopia (30), a national survey in Egypt (43), China (39), and also a study done in the United States (35). The reason behind this might be as the age increase the susceptibility to infectious disease will also increase (44, 45). Another reason might be as age increases the chance of having comorbid chronic disease also increased which put them at high risk to be infected with the pandemic, and it influences them to have the willingness to take the COVID-19 vaccine.

Compared to HCWs who have poor attitudes, those who have a good attitude were more likely to accept COVID-19. This finding was similar to the study finding in the Hospitals of South Gondar Zone, Ethiopia (30), southwestern Ethiopia (6), and the Democratic Republic of the Congo (37). The possible reason might be a good attitude toward the vaccine might avoid misinformation, misunderstanding, and misconception toward the vaccine and outweigh its importance and then improves their willingness to accept the COVID-19 vaccine.

Another important finding was that significant differences were observed between the intention to get vaccinated and vaccination beliefs. HCWs who had higher perceived susceptibility to COVID-19 were more likely to accept the COVID-19 vaccine than their counterparts. This finding was supported by the study done in South Gondar Zone Hospitals, Ethiopia (30), and a study done in China (39). This might be due to the fact that the Health Belief Model is known to predict intention to receive the COVID-19 Vaccine (22, 33, 46, 47). In addition, because HCWs are involved in the treatment of patients they consider themselves at a higher risk of infection than others and are motivated to get vaccinated in need to build their immunity by taking the COVID-19 vaccine (48).

The likelihood of HCWs accepting the COVID-19 vaccine is more predicted by the higher perceived severity and seriousness of COVID-19 disease compared to their counterparts. This finding was supported by a study done among HCWs in China (39). The possible reasons are individuals who perceive the COVID-19 disease as severe and serious might choose to be vaccinated (49). Again, it's known that the health belief model (HBM) is a widely used model in vaccination behavior, particularly COVID- 19. The likelihood of an individual adopting a particular health behavior (e.g., getting the COVID-19 vaccine) is determined by the perceived susceptibility and severity of illness or disease (e.g., COVID-19), along with the belief in the effectiveness of the recommended health behavior (e.g., COVID-19 vaccination) (50). In addition, when there is perceived severity, stress was experienced and HCWs were more initiated to have the willingness to take the COVID-19 vaccine (51).

## Conclusion

The Willingness to accept the COVID-19 vaccine among study participants was low. Factors significantly associated with the willingness to accept the COVID-19 vaccine were age, good attitude, perceived susceptibility, and perceived severity/seriousness of the disease. The low willingness of healthcare workers to accept the COVID-19 vaccine was alarming to mitigate the transmission of the pandemic from the clients to providers and vice-versa. It needs more emphasis from the government in collaboration with other stakeholders to address the concerns and provide reliable information to avert misconceptions and rumors about the vaccine to improve the vaccine status of healthcare workers to protect the communities.

### Strength and limitation

The strength of this study was data collection tool used for this study was different and modified from the previous study (we used the health belief model measures) to assess the HCWs' willingness to accept the COVID-19 vaccine. In addition, Cronbach's α of the HBM domain items were above the accepted standard criteria (showed a high internal consistency). The present study has several limitations. First, acceptance of getting the COVID-19 vaccine was self-reported by participants, and hence the information bias probably existed in this study. Second, this was a cross-sectional survey based on self-reported information; hence, causality inference can hardly be drawn, using a cross-sectional study design to show only a temporal link between exposure and outcome variables. Third, this study doesn't show that vaccine acceptance changes over time because of the ever-changing HCWs' perception of risk for COVID-19 and information related to vaccination safety and efficacy.

## Data availability statement

The original contributions presented in the study are included in the article/supplementary material, further inquiries can be directed to the corresponding author/s.

## Ethics statement

The studies involving human participants were reviewed and approved by Haramaya University's Institutional Health Research Ethics Review Committee (IHRERC), College of Health and Medical Sciences (ref. number. IHRERC/069/2021), provided ethical approval for this study. The patients/participants provided their written informed consent to participate in this study.

## Author contributions

TG and AS: conceptualization, methodology, writing—original draft, investigation, project administration, and analysis. ML, AE, BB, AD, BE, MD, SM, AN, HB, GT, DT, KN, HA YD, and AA: conceptualization, methodology, data collection, and writing — review and editing. Moreover, the co-authors wrote the manuscript. All authors were involved in reading and approving the final manuscript.

## Conflict of interest

The authors declare that the research was conducted in the absence of any commercial or financial relationships that could be construed as a potential conflict of interest.

## Publisher's note

All claims expressed in this article are solely those of the authors and do not necessarily represent those of their affiliated organizations, or those of the publisher, the editors and the reviewers. Any product that may be evaluated in this article, or claim that may be made by its manufacturer, is not guaranteed or endorsed by the publisher.

## Abbreviations

AOR: Adjusted Odd Ratio
CI: Confidence interval
COR: Crude Odd Ratio
COVID-19: Coronavirus disease in the year 2019
EDHS: Ethiopian Demographic and Health Survey
FOMH: Federal Ministry of Health
HCW: Health Care Workers
USAID: United States Agency for International Development
WHO: World Health Organization.
